# Modeling the potential distribution of different types of *Dendrocalamus sinicus,* the strongest woody bamboo in the world, with MaxEnt model

**DOI:** 10.7717/peerj.13847

**Published:** 2022-08-02

**Authors:** Peitong Dou, Yuran Dong, Lingna Chen, Han-Qi Yang

**Affiliations:** 1 Institute of Highland Forest Science, Chinese Academy of Forestry, Kunming, Yunnan, China; 2 College of Landscape Architecture, Nanjing Forestry University, Nanjing, Jiangsu, China; 3 College of Biology and the Environment, Nanjing Forestry University, Nanjing, Jiangsu, China

**Keywords:** *Dendrocalamus sinicus*, key bioclimate variables, MaxEnt model, Potential distribution area, Woody bamboo

## Abstract

Climate change will significantly affect the distribution area of species. Through establishing distribution model, we can simulate the current and future potential distribution range and provide reference for the introduction and cultivation planning of rare or economic plants. *Dendrocalamus sinicus*, endemic to Yunnan Province of China, is the strongest woody bamboo in the world. In the present study, the MaxEnt model was performed to simulate the distribution of different types of *D. sinicus* in China and neighboring countries or regions. The results suggested that the suitable distribution range of “straight type”, the main type for cultivation and utilization, was 8°–30°N and 73°–122°E under the current climate conditions, while the potential distribution range of “bending type” was 6°–31°N and 79°–109°E. The two most key climate variables associated with distribution of “straight type” were “Temperature Annual Range” with 36.6% contribution rate and “Temperature Seasonality” (32.4%), while “Isothermality” (47.8%) and “Precipitation of Driest Month” (24.8%) for “bending type”. Under different climate change scenarios (SSP1-2.6, SSP5-8.5) and periods (2050, 2090), the potential distribution area of the “straight type” were apparently different, indicating that the distribution area of *D. sinicus* will be affected significantly by climate changes in the future. Our findings would be not only beneficial to understanding limiting factors for natural distribution of *D. sinicus*, but also helpful for further germplasm conservation, introduction and cultivation planning of this rare woody bamboo.

## Introduction

Nowadays, global warming and biodiversity conservation are some of the most crucial challenges to human sustainable development, and the impacts of climate change on species survival have become hot-spot issue accordingly (Harrington, Fleming & Woiwod, 2001; Kriegler et al., 2017; Wu et al., 2018). In general, the global climate change significantly affects the distribution range of species, especially for species sensitive to climate factors (Atwater & Barney, 2021). Therefore, the study on species distribution shift under the background of climate change will be beneficial to the conservation and utilization of biological germplasm resources (Liu et al., 2022). At present, many models for predicting the potential distribution of species were developed, such as bioclim (bioclimatic prediction system) (Honig, Cowling & Richardson, 1992), domain (domain model) (Carpenter, Gillison & Winter, 1993), GARP (genetic algorithm for rule set prediction) (Anderson, Lew & Peterson, 2003), Enfa (ecological niche factor analysis) (Engler, Guisan & Rechsteiner, 2010) and the MaxEnt (maximum entropy approach) model (Phillips, Anderson & Schapire, 2006). Among them, the MaxEnt model was widely used in predicting the change trend of distribution area of the endangered or rare species (*e.g.*, Goldenberg, Reginato & Michelangeli, 2020; Peng et al., 2019; Trisurat et al., 2013), and the impact of climate change on species distribution and ecosystems (*e.g.*, Iannella et al., 2021; Nabout et al., 2016; Noulèkoun et al., 2017). In the above researches, the MaxEnt model have exhibited its advantages, such as short running time, small sample size required, high accuracy, and Jackknife test to evaluate the contribution rates of various environmental variables in the model (Li et al., 2019b; Saupe et al., 2015). At present, the potential distribution of many rare or economically important plants has been predicted using the MaxEnt model (*e.g.*, Peng et al., 2019; Yi et al., 2016; Zhang et al., 2016), but there were few studies on the distribution model of rare woody bamboos.

Woody bamboo belongs to the bamboo subfamily of Gramineae (Poaceae), with about 80 genera and more than 1,500 species (Yi et al., 2008; Soreng et al., 2017). Under natural conditions, it is distributed in continents except for Europe and Antarctica, its species diversity is concentrated in the tropical and subtropical regions of Asia, Africa and South America (Yi et al., 2008). Yunnan Province of China, one of the modern distribution centers of bamboos in the world, possesses more than 220 native bamboo species from 28 genera, is known as “the hometown of bamboo in the world” (Hui, Yang & Du, 2006). Remarkably, *Dendrocalamus sinicus,* endemic to southern and southwestern Yunnan, is the largest woody bamboo species documented in the world. It is a subtropical and tropical sympodium bamboo with a diameter at breast height (DBH) of 30 cm and a height of nearly 30 m. The peak period of bamboo shooting is from July to August, depending on the beginning date of rainy season (Hui, Yang & Du, 2006; Guo, Chen & Yang, 2019). The average wet weight of culm is 100–150 kg, and the culm timber yield per unit areas is 5–8 times more than Moso bamboo (*Phyllostachys edulis*), which is the main economic bamboo species in eastern Asia (Hui, Yang & Du, 2006). Therefore, *D. sinicus* has great development potential in timber, paper making and handicraft industry, *etc*. As a precious and rare bamboo species in the world, it is of high scientific research, economic and cultural value (Hui, Yang & Du, 2006).

**Figure 1:**
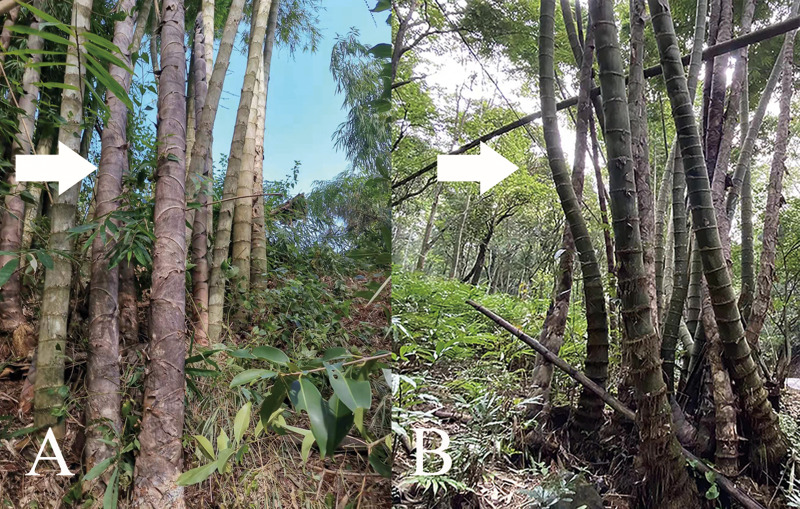
Morphological characteristics of straight type (A) and bending type (B). Photographs by Peitong Dou.

Within the distribution range of *D. sinicus*, two natural types of culm, namely “straight type” and “bending type”, are detected (Gu et al., 2012; Yang et al., 2018; Guo, Chen & Yang, 2019). The “straight type” bears straight and terete culms from head to foot, and its internodes are normal and smooth (Fig. 1A). It is not only the excellent raw material for timber and building materials, but also the main type of industrial utilization and cultivation (Hui, Yang & Du, 2006). On the other hand, the “bending type” has abnormal culms at the lower half (Fig. 1B), and its internodes are swollen, shortened or deformed, which is a little value in garden landscaping. The significant differences in ecological conditions were detected between the habits of two types (Hui, Yang & Du, 2006). The “straight type” usually distributes at subtropical mountains with 1,000–1,500 m above sea level, while the “bending type” occurs in habitats of marginal tropical montane regions at elevations of ca. 500–1,000 m (Gu et al., 2012; Yang et al., 2018).

So far, due to the integrative influence of climate change and biological characteristics, *e.g.*, sporadic flowering and low seed setting rate, the habitat of *D. sinicus* is scattered and high-quality germplasm resources are scarce (Gu et al., 2012; Xie et al., 2019). Therefore, it is necessary to choose suitable areas to protect and propagate this rare bamboo species. In the present study, we use the MaxEnt model to screen the key environmental factors affecting the distribution of *D. sinicus,* and to predict potential distribution areas. Our aims are to distinguish out the key natural factors limiting the distribution of *D. sinicus,* and to provide a scientific basis for its germplasm conservation and further introduction and cultivation planning.

## Materials & Methods

### Species data sources

**Figure 2:**
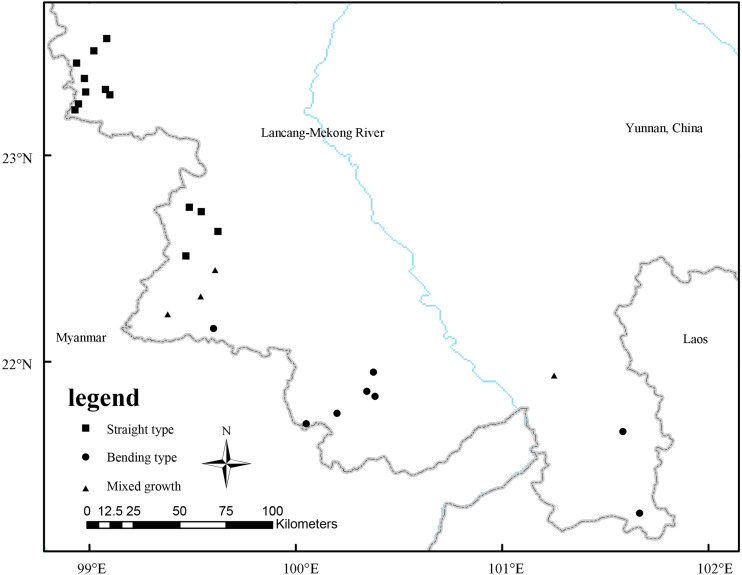
Spatial distribution of occurrence records of *D. sinicus* in China.

By searching the Chinese Virtual Herbarium (https://www.cvh.ac.cn/), National Science and Technology Infrastructure (http://www.nsii.org.cn/2017/home.php), Flora Reipublicae Popularis Sinicae (http://www.iplant.cn/frps), Global Biodiversity Information Facility (http://www.gbif.org) as well as our investigation and sample collection in the field, a total of 25 distribution locations of *D. sinicus* were obtained (Table 1) after checking the integrity of coordinate information and eliminating the duplicate coordinates. In the previous molecular genetic study (Yang et al., 2018), all existing populations of *D. sinicus* were divided into two genotypes, *i.e.*, “straight type” and “bending type”, which was consistent with the morphological characters of culms (Gu et al., 2012). Therefore, according to the previous results, 25 coordinate points were divided into 13 “straight type”, eight “bending type” and four mixed coordinate points. All coordinates were saved in the CSV (comma-separated values) format for MaxEnt model analysis according to culm type. Software ArcGIS 10.2 was used to reproduce the current distribution points of *D. sinicus* (Fig. 2).

**Table 1:** Occurrence records of *D. sinicus* in China.

Code	Longitude	Latitude	Culm type
1	99.02056	23.50694	Straight type
2	98.93806	23.44833	Straight type
3	98.97528	23.37417	Straight type
4	99.07778	23.32	Straight type
5	98.98111	23.30889	Straight type
6	99.09944	23.295	Straight type
7	98.94722	23.25111	Straight type
8	98.92972	23.22139	Straight type
9	99.54139	22.72806	Straight type
10	99.62222	22.63278	Straight type
11	99.4675	22.51417	Straight type
12	99.08333	23.56667	Straight type
13	99.48333	22.75	Straight type
14	99.60861	22.44333	Mixed growth
15	99.53861	22.31639	Mixed growth
16	99.37944	22.22917	Mixed growth
17	101.2517	21.93306	Mixed growth
18	99.60139	22.16111	Bending type
19	100.3444	21.85778	Bending type
20	100.377	21.94987	Bending type
21	101.5849	21.66172	Bending type
22	101.6667	21.26667	Bending type
23	100.2	21.75	Bending type
24	100.05	21.7	Bending type
25	100.3833	21.83333	Bending type

### Climate variables

In this study, bioclimatic variables from the WorldClim website (https://www.worldclim.org/data/worldclim21.html#google_vignette) (Table S1) was used in further analysis. We removed variables 8, 9, 18, and 19 because of spatial artifacts (Ashraf et al., 2017). The environmental variables, recorded from 1970 to 2000, were saved in file format of ASCII (American Standard Code for Information Interchange) converted by ArcGIS 10.2, and were used to establish the initial model (Fick & Hijmans, 2017). In order to eliminate the auto-correlation and colinearity between variables, the Pearson correlation coefficient (r) and principal component analysis (PCA) of 15 bioclimate variables from occurrence records were tested, and only one variable from each set of highly cross-correlated variables (—r— > 0.8) was kept for further analysis according to Yi et al. (2016). For instance, the variables bio6 was correlated with both bio5 (*r* = 0.8) and bio7 (*r* = − 0.9), then bio6 was dropped and both bio5 and bio7 were reserved based on the PCA result.

Future climate projections were extracted from the Shared Socioeconomic Pathways (SSPs) of the BCC-CSM2-MR global climate (2021–2040, 2041–2060, 2061–2080, and 2081–2100) database in the Coupled Model Intercomparison Projects 6 (CMIP6) (https://www.worldclim.org/data/cmip6/cmip6_clim2.5m.html). The associated scenarios included the SSP1-2.6 (ssp126), SSP2-4.5 (ssp245), SSP3-7.0 (ssp370), and SSP5-8.5 (ssp585), following Liu et al. (2021a). The SSPs reflect four different developments of the world that were characterized by varying levels of global challenges. We selected SSP1-2.6 (low forcing scenario, radiation intensity reaches 2.6w/m^2^ in 2100) and SSP5-8.5 (high forcing scenario, radiation intensity reaches 8.5 w/m^2^ in 2100) as future test scenarios according to Zhang, Chen & Xin (2019), and simulated the suitable distribution of “straight type” in the 2050s (2041–2060) and 2090s (2081–2100). All environmental data used in this model were 2.5-arc minute spatial resolution (also referred to as 4.5 km spatial resolution).

### Construction and test of the MaxEnt model

For each type, we created 493 candidate models by combining 17 values of regularization multiplier (0.1–1.0 at intervals of 0.1, 2–6 at intervals of 1, as well as 8 and 10), and all 29 possible combinations of five feature classes (linear = L, quadratic = Q, product = P, threshold = T, and hinge = H). The performance of the candidate model was evaluated on the basis of significance (partial ROC, with 500 iterations and 50 percent of data for bootstrapping), omission rates (E = 5%), and model complexity (Akaike Information Criterion corrected for small sample sizes, AICc). The best models were selected according to the significant models and the omission rate ≤ 5%. The models of Delta AICc ≤ 2 were selected as final models in this model set (Cobos et al., 2019; Alkishe & Peterson, 2022). The complete set of occurrences and the selected parameterizations were used to create the final models for the two types. We produced 10 replicates by bootstrap, with logistic outputs, and transferred these models to the 12 countries or regions for current and future scenarios. Partial ROC and omission rates (E = 5%) were evaluated for the final models (Cobos et al., 2019). The above analysis was performed through the kuenm R package (https://github.com/marlonecobos/kuenm). The receiver operating characteristic curve (ROC) of all environmental variables was calculated, and the importance of different environmental variables was measured by Jackknife test, following Li et al. (2019a). The areas under the receiver operating characteristic curve (AUC) were used to evaluate the accuracy of the model (Goldenberg, Reginato & Michelangeli, 2020). The evaluation criteria are as follows: 0.5 < AUC ≤ 0.6 (the model is failed), 0.6 < AUC ≤ 0.7 (“poor”), 0.7 < AUC ≤ 0.8 (“general”), 0.8 < AUC ≤ 0.9 (“better”) and 0.9 < AUC ≤ 1 (“excellent”). The closer the AUC value was to 1, the higher was the prediction accuracy of the model (Wang et al., 2018; Phillips et al., 2009). The suitability maps were calculated using the logistic output of MaxEnt, which ranges from 0 (lowest suitability) to 1 (highest suitability). For visualization and further analysis, the prediction results were imported into ArcGIS 10.2 and divided the habitat suitability maps into four levels according to expert experience and relevant literature (Hui, Yang & Du, 2006; Zhang et al., 2019): unsuitable habitat (0–0.35), poorly suitable habitat (0.35–0.55), moderately suitable habitat (0.55–0.75), and highly suitable habitat (0.75–1).

## Results

### Model selection and accuracy evaluation

After kuenm R package screening, the feature class was LP and regularization multiplier was 2 in the final model parameter combination of the “straight type”. As for the “bending type”, the feature class was LQTH and regularization multiplier was 3. We rebuild the model using the optimized parameter combination. The AUC values of the “straight type” and “bending type” were 0.978 and 0.996 (Fig. S1) respectively, indicating both models were “excellent”. The above results suggested that the distribution area simulation using the MaxEnt model was reliable, and we further analyzed the impact of climate change on the distribution area of *D. sinicus*.

### Selection of key climatic factors

For each type of *D. sinicus*, four most key climate variables affecting the geographical distribution were screened out using the MaxEnt model. As for the “straight type”, four key climate variables with a cumulative contribution rate of 94.8% were: Temperature Annual Range (bio7; 36.6%), Temperature Seasonality (bio4; 32.4%), Annual Precipitation (bio12; 15.9%) and Precipitation of Wettest Month (bio13; 9.9%) (Table 2). On the other hand, four key climate variables of the “bending type” were the Isothermality (bio3; 47.8%), Precipitation of Driest Month (bio14; 24.8%), Temperature Seasonality (bio4; 16.5%) and Mean Temperature of Coldest Quarter (bio11; 8.7%) (Table 2), with a cumulative contribution rate of 97.8%. The results of Pearson correlation coefficient (r) analysis indicated that none of correlation coefficient between the environmental variables exceeded 0.8 (Table 3). Because altitude and solar radiation were highly correlated with many climate variables (Tables S2, S3), climate variables were preferentially selected as variables for the classification of suitable areas, rather than solar radiation and altitude. Finally, all four climate variables above were deemed as the main factors affecting the distribution of two types. On this basis, the MaxEnt models of the “straight type” and “bending type” distributions were established and were further evaluated.

**Table 2:** The dominant factors affecting the potential distribution of different culm types for *D. sinicus*.

Category	Bioclimatic variables	Abbreviation	Percent contribution	Accumulated Percent contribution/%
	Temperature Annual Range	bio7	36.6	36.6
Straight type	Temperature Seasonality (standard deviation *100)	bio4	32.4	69
	Annual Precipitation	bio12	15.9	84.9
	Precipitation of Wettest Month	bio13	9.9	94.8
	Isothermality	bio3	47.8	47.8
Bending type	Precipitation of Driest Month	bio14	24.8	72.6
	Temperature Seasonality (standard deviation *100)	bio4	16.5	89.1
	Mean Temperature of Coldest Quarter	bio11	8.7	97.8

**Table 3:** Pearson correlation coefficient of key environmental factors affecting the distribution of different culm types for *D. sinicus*.

	Code	bio7	bio4	Bio12	bio13
	bio7	1			
Straight type	bio4	0.712	1		
	bio12	−0.590	−0.235	1	
	bio13	0.180	0.617	0.458	1
	**Code**	**bio3**	**bio14**	**bio4**	**bio11**
	bio3	1			
Bending type	bio14	0.215	1		
	bio4	−0.749	−0.188	1	
	bio11	0.150	0.261	−0.142	1

**Notes.**

** The difference is very significant at the level of 0.01.

### Relationship between geographical distribution and environmental variables

The importance values of key climate variables to the distribution of *D. sinicus* were analyzed using the Jackknife test in the MaxEnt model. Within the “straight type”, the order of the four key climate factors was Temperature Seasonality (bio4), Temperature Annual Range (bio7), Annual Precipitation (bio12) and Precipitation of Wettest Month (bio13) (Fig. S2), based on importance. And their appropriate variation ranges were <390, <23.9 °C, >1,418 mm and > 282 mm, respectively (Table 4). As for the “bending type”, the order of four key climate factors was Temperature Seasonality (bio4), Isothermality (bio3), Mean Temperature of Coldest Quarter (bio11) and Precipitation of Driest Month (bio14) (Fig. S2), and their appropriate variation ranges (optimal values) were 210–410 (338), > 49.1, 10.7–20.4 (15.9) °C and 7.2–34.0 (13.2) mm, respectively (Table 4). The response curves of environmental variables to the distribution probability were shown in Figs. S3 and S4. When the environmental variables were lower than the optimal value, the distribution probability increased with the increase of the environmental variables. And it worked in the reverse too.

**Table 4:** Suitable range of environmental variables of different culm types for *D. sinicus*.

Category	Environmental variables (abbreviation)/Unit	Suitable range (Optimum value)
	Temperature Annual Range (bio7)/°C	<23.9
Straight type	Temperature Seasonality (standard deviation *100) (bio4)	<390
	Annual Precipitation (bio12)/mm	>1418
	Precipitation of Wettest Month (bio13)/mm	>282
	Isothermality (bio3)	>49.1
Bending type	Precipitation of Driest Month (bio14)/mm	7.2–34.0 (13.2)
	Temperature Seasonality (standard deviation *100) (bio4)	210–410 (338)
	Mean Temperature of Coldest Quarter (bio11)/°C	10.7–20.4 (15.9)

### The suitable areas of different types under current climatic scenario

**Figure 3:**
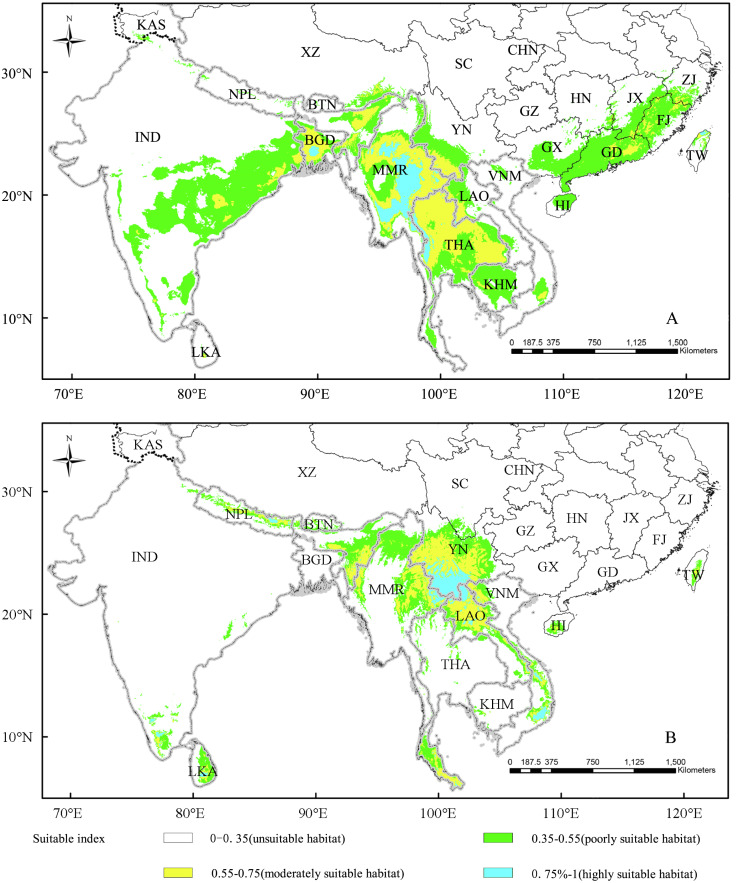
The suitability distribution area of straight type (A) and bending type (B) under the current scenario. Vietnam-VNM, Thailand-THA, Nepal-NPL, Myanmar-MMR, Sri Lanka-LKA, Laos-LAO, Cambodia-KHM, India-IND, Bhutan-BTN, Bangladesh-BGD, China-CHN, Kashmir-KAS, Tibet-XZ, Sichuan-SC, Yunnan-YN, Guizhou-GZ, Guangxi-GX, Guangdong-GD, Hainan-HI, Fujian-FJ, Zhejiang-ZJ, Jiangxi-JX, Hunan-HN, Taiwan-TW.

MaxEnt model predicted the potentially suitable areas of *D. sinicus* included 12 countries or regions under the current environmental conditions. The potential distribution range of the “straight type” was between 8°–30°N and 73°–122°E (Fig. 3A), and the distribution area covered 2234583.37 km^2^ (Table 5). Among potential distribution range, the countries with large distribution areas were India (30.86%), China (24.61%), Myanmar (16.72%), and Thailand (15.86%) (Table 5). In addition, the highly suitable areas, with a cumulative area of 133,072.92 km^2^, were mainly distributed in Myanmar (81.57%), Thailand (12.82%), and Bangladesh (3.94%) (Table 5). By and large, the suitable areas of the “straight type” extended along the longitude and latitude from the existing distribution range, and the extended range along the longitude was much larger than that of the latitude.

**Table 5:** Predicted potential distribution area for straight type under current climatic conditions.

Country	Unsuitable habitat(km^2^)	Poorly suitable habitat(km^2^)	Moderately suitable habitat(km^2^)	Highly suitable habitat(km^2^)	Suitable habitat (km^2^)	Percentage of highly suitable areas in total(%)	Percentage of suitable areas in total(%)
Vietnam	249,045.14	24,149.31	3,524.31	0.00	27,673.61	0.00	1.24
Thailand	76,458.33	121,562.50	215,885.42	17,065.97	354,513.89	12.82	15.86
Nepal	129,600.70	6024.31	0.00	0.00	6024.31	0.00	0.27
Myanmar	205,625.00	100,086.81	165,052.09	108,541.67	373,680.56	81.57	16.72
Sri Lanka	52,500.00	642.36	572.92	0.00	1215.28	0.00	0.05
Laos	126,718.75	46,475.70	23,298.61	451.39	70,225.70	0.34	3.14
Cambodia	65,173.61	81,753.47	3,628.47	0.00	85,381.95	0.00	3.82
India	1,930,017.39	626,458.34	63,229.17	0.00	689,687.51	0.00	30.86
Bhutan	33,836.81	416.67	0.00	0.00	416.67	0.00	0.02
Bangladesh	48,836.81	29,357.64	38,802.08	5,243.06	73,402.78	3.94	3.28
China	9,078,402.92	470,017.37	78,142.36	1,770.83	549,930.56	1.33	24.61
Kashmir	179,583.34	2,152.78	277.78	0.00	2,430.56	0.00	0.11
Total	12,175,798.81	150,9097.25	592,413.20	133,072.92	2,234,583.37	100.00	100.00

As for the “bending type”, its potential distribution range was between 6°–31°N and 79°–109°E (Fig. 3B), and the highly suitable areas were mainly distributed in southwest China, northern Laos and eastern Myanmar. Different from the “straight type”, the distribution range of the “bending type” mainly displayed a trend of southward expansion along low latitude to the tropics. It was worth noting that the potential distribution areas of the two variants were significantly larger than the existing ranges.

**Figure 4:**
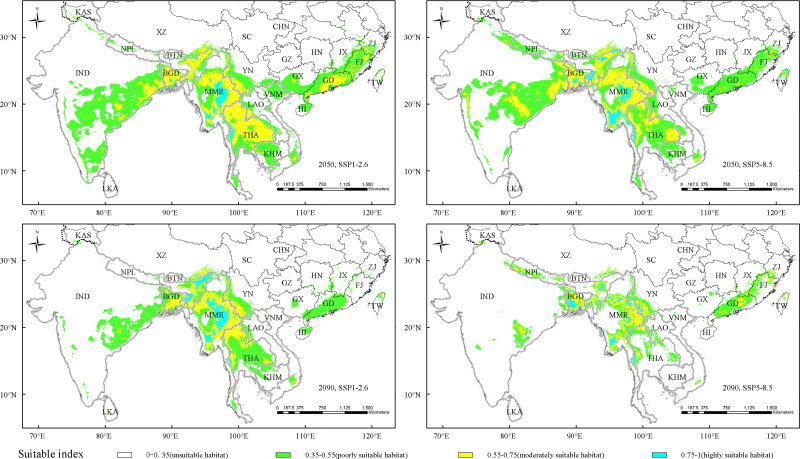
Potentially suitable climatic distribution of straight type under different climate change scenarios. Vietnam-VNM, Thailand-THA, Nepal-NPL, Myanmar-MMR, Sri Lanka-LKA, Laos-LAO, Cambodia-KHM, India-IND, Bhutan-BTN, Bangladesh-BGD, China-CHN, Kashmir-KAS, Tibet-XZ, Sichuan-SC, Yunnan-YN, Guizhou-GZ, Guangxi-GX, Guangdong-GD, Hainan-HI, Fujian-FJ, Zhejiang-ZJ, Jiangxi-JX, Hunan-HN, Taiwan-TW.

### The suitable distribution area of the “straight type” under future climate scenarios

As an excellent timber bamboo species and main type for industrial utilization and cultivation, the “straight type” is our concerning focus, and in this study, we predicted the potentially suitable distribution area of the “straight type” under SSP1-2.6 and SSP5-8.5 future climate change scenarios (Fig. 4). Under the SSP1-2.6 scenario in 2050, the highly suitable habitat of the “straight type” was mainly in Myanmar and western Thailand. While under SSP5-8.5 in 2050 and SSP1-2.6 in 2090, the highly suitable habitat basically disappeared in western Thailand, but emerged in parts of eastern India. In addition, under SSP5-8.5 in 2090, the suitable habitat, especially for the highly suitable distribution, sharply shrank in eastern India, southern China, Cambodia, Thailand and Myanmar.

Compared with the current distribution, three types of the predicted suitable habitats, namely poorly, moderately and total highly suitable regions, exhibited different change trends under the climate change scenarios SSP1-2.6 and SSP5-8.5 in 2050. The total highly suitable regions displayed a decreasing trend, namely 27.42% and 8.19% under SSP1-2.6 and SSP5-8.5 scenarios respectively (Table 6). But the poorly, moderately and total suitable habitat had an increasing trend, and the increase rate of suitable area under low radiation intensity (SSP1-2.6) was higher than that of high radiation intensity (SSP5-8.5).

**Table 6:** Potentially suitable climatic distribution of straight type under different climate change scenarios.

Decades	Scenarios	Predicted area/10^4^km^2^				Increase/decrease rate (%) [Compared to the current distribution]			
		Total poorly suitable habitat	Total moderately suitable habitat	Total highly suitable habitat	Totalsuitable habitat	Total poorly suitable habitat	Total moderately suitable habitat	Total highly suitable habitat	Total suitable habitat
Current	–	150.91	59.24	13.31	223.46	–	–	–	–
2050s	ssp126	162.50	80.91	9.66	253.07	7.68	36.59	−27.42	13.25
	ssp585	151.17	71.57	12.22	234.96	0.17	20.81	−8.19	5.15
2090s	ssp126	97.66	44.29	13.39	155.33	−35.29	−25.24	0.59	−30.49
	ssp585	55.66	31.25	8.31	95.23	−63.11	−47.25	−37.55	−57.38

On the other hand, comparison with the current distribution, almost predicted suitable habitats under two climate scenarios in 2090 exhibited dramatically decreasing trends, except the highly suitable area slightly increased 0.59% under the SSP1-2.6 climate scenario in 2090 (Table 6). Meanwhile, the high radiation intensity scenarios (SSP5-8.5) had a much stronger effect on decreasing suitable habitat than that of low radiation intensity scenarios (SSP1-2.6). The reduction of suitable areas under SSP5-8.5 in 2090, to a large extent, were due to the exclusion of eastern India, southern China (GX, HI, GD, FJ and JX), Cambodia and Thailand (Fig. 4).

## Discussion

### Key climate factors determining the survival and distribution of *D. sinicus*

As the strongest woody bamboo documented in the world, *D. sinicus* has outstanding performance in producing timber. Meanwhile, because of narrow distribution and provenance scarcity, its germplasm conservation and introduction planning have raised extensive attentions since publication as a new species (Chia & Sun, 1982; Hui, Yang & Du, 2006; Gu et al., 2012). The consequent problem is to clarify the key climate factors determining the survival and distribution of *D. sinicus,* which is important but yet controversial. Empirically, low temperature in winter (Hui, Yang & Du, 2006) or precipitation (Pu, 2004) were once considered to be the most key climate variable determining the growth and distribution of *D. sinicus*. In this study, the MaxEnt model detected that the bioclimatic variables of the highest contribution rate to the distribution of two types were different, namely Temperature Annual Range (36.6%) for the “straight type” and Isothermality (47.8%) for the “bending type” respectively (Table 2), indicating that temperature was probably the most key factor affecting the survival of *D. sinicus*. This result was similar to opinion of Hui, Yang & Du (2006). It may be due to the biological characteristics of *D. sinicus*, namely, it is a tropical bamboo species occurred at the edge of the tropics, and the low temperature in winter will seriously threaten its survival (Chia & Sun, 1982; Hui, Yang & Du, 2006).

### Suitable distribution range based on the MaxEnt model and introduction practice of *D. sinicus*

According to the prediction of the MaxEnt model, the current suitable distribution ranges of the two types were different. For the “straight type”, the suitable distribution range was 8°–30°N and 73°–122°E with a broad span of east–west distribution, while the potential distribution range of “bending type” was 6°–31°N and 79°–109°E. Compared with the “straight type”, the highly suitable habitat of “bending type” appeared sporadically in south Vietnam, south India and Sri Lanka, This suggested that the distribution range of “bending type” exhibited a southward spreading trend to the tropical area, which was similar to the previous research results (Yang et al., 2018). The identical environmental variable affecting the distributions of the two types was Temperature Seasonality, which is the most important variables in the Jackknife test, and the maximum value of Temperature Seasonality in the “bending type” was higher than that in the “straight type” (Table 4). This might reflect that the “bending type” could survive in regions with larger temperature seasonality variation, and had more tropical attribute than “straight type” (Chia & Sun, 1982; Hui, Yang & Du, 2006; Yang et al., 2018).

On the aspect of introduction and cultivation planning of *D. sinicus*, our results also provided some new views. Conventionally, planning for plant introduction and cultivation range was mainly based on similar climate, soil conditions and other factors between the introduction area and the origin habitat (Hui, Yang & Du, 2006). The previous study inferred that the climate conditions of the most suitable area for the “straight type” were as follows: (1) the average temperature in the coldest month was ≥13 °C; (2) the days of the daily minimum temperature ≤ 0 °C were not more than 1 day; (3) frost did not happen throughout the year; and (4) the annual precipitation was ≥ 1200 mm (Hui, Yang & Du, 2006). From 2000 to 2003, the scientists introduced and cultivated “straight type” at 12 predicted suitable areas in eight counties of Yunnan Province (Hui, Yang & Du, 2006; An & Chen, 2010). After 15 years, the introduced bamboo clumps of *D. sinicus* survived only at two sites: Ning’er County (23°32′N) and Xinping County (24°04′N), in which clumps could bear new shoots normally and the culms grew up to 20 cm in diameter. Moreover, approximate 10% introduced clumps bloomed and died in 1–2 years after cultivating. The above results suggested the strict conditions for the introduction and cultivation of *D. sinicus*. On the other hand, based on the MaxEnt model, we predicted the optimal climate conditions of highly suitable areas for “straight type” were: (1) the Temperature Annual Range was <23.9 °C; (2) the Precipitation of Wettest Month was >282 mm and (3) the Annual Precipitation was >1,418 mm, respectively. Compared with the empirical climate variables for highly suitable areas of “straight type”, our climate conditions based on MaxEnt model were more strict. However, our results were also more consistent with the actual outcomes, indicating our results had a higher reliability.

### Change trend on suitable habitat of “straight type” under different climate change scenarios

The adaptation to climate and climate change is vital to plant growth, geographical distribution and biodiversity (Liu et al., 2022; Lv & Wu, 2009; Yi et al., 2017). In the present study, the potential suitable distribution area of “straight type”, predicted by MaxEnt model, were different under two climate change scenarios (Table 6). Compared with the current distribution, the total suitable habitat of “straight type” would increase in 2050 and decrease in 2090 under same radiation intensity. Furthermore, the total suitable habitat would increase slightly (2050) and decrease dramatically (2090) under high radiation intensity (SSP5−8.5) (Table 6), implying that higher radiation intensity (SSP5−8.5) would limit distribution of the “straight type”. The previous studies indicated that global warming would increase, fluctuate or decrease the distribution range of species (Thomas et al., 2004; Yuan, Wei & Wang, 2015). Our results also suggested that the impact of climate change on plant distribution might be a long-term process. The slow increase of radiation intensity in a short period would not give rise to significant changes in distribution area of species. This is because plants possess certain capacity of self-regulation and diffusion (Liu et al., 2021b), which result in fluctuated suitable distribution area. In extreme cases, for example, excessive radiation intensity will affect the growth and development of plants, and eventually lead to death. In turn, the distribution area of plants will reduce or disappear (Li, Fan & He, 2020).

In summary, our results confirmed that the “straight type” had poorer heat resistance than “bending type”, which is important to instruct future introduction and cultivation planning of *D. sinicus.* In addition, we should also realize that the MaxEnt model does not consider the factors such as soil and soil microorganisms at specific sites, so the accuracy of this prediction is limited.

## Conclusions

Based on *D. sinicus* occurrence records and bioclimatic variables, the current and future suitable habitat of *D. sinicus* in China and adjacent regions was modeled using MaxEnt model for the first time. The bioclimatic variables of temperature annual range and isothermality were revealed to have crucial effect on *D. sinicus* distribution. In the next 70 years, the habitat suitability of this woody bamboo may be different with climate change. The prediction of this study is of strategic significance for further germplasm conservation, introduction and cultivation planning of this rare and precious bamboo species.

## Supplemental Information

10.7717/peerj.13847/supp-1 Supplemental Information 1 ROC curve and AUC value

10.7717/peerj.13847/supp-2 Supplemental Information 2 Jackknife test of variable importance

10.7717/peerj.13847/supp-3 Supplemental Information 3 Response curves of environmental variables to distribution probability for straight type

10.7717/peerj.13847/supp-4 Supplemental Information 4 Response curves of environmental variables to distribution probability for bending type

10.7717/peerj.13847/supp-5 Supplemental Information 5 19 bioclimatic variables

10.7717/peerj.13847/supp-6 Supplemental Information 6 The correlation analysis between climate factors, altitude and solar radiation from bending coordinate points

10.7717/peerj.13847/supp-7 Supplemental Information 7 The correlation analysis between climate factors, altitude and solar radiation from straight coordinate points

10.7717/peerj.13847/supp-8 Supplemental Information 8 Bending type raw data

10.7717/peerj.13847/supp-9 Supplemental Information 9 Straight type raw data

## Additional Information and Declarations

### Data Availability

The following information was supplied regarding data availability: Raw data is available in the Supplementary Files.
