# Supplementary material for: Modeling the potential distribution of different types of Dendrocalamus sinicus, the strongest woody bamboo in the world, with MaxEnt model
Source: PeerJ. 2022 Aug 2;10:e13847. doi: 10.7717/peerj.13847 (PMC9354798; doi:10.7717/peerj.13847)
Supplement: Supplemental Information 1 [file peerj-10-13847-s001.pdf]

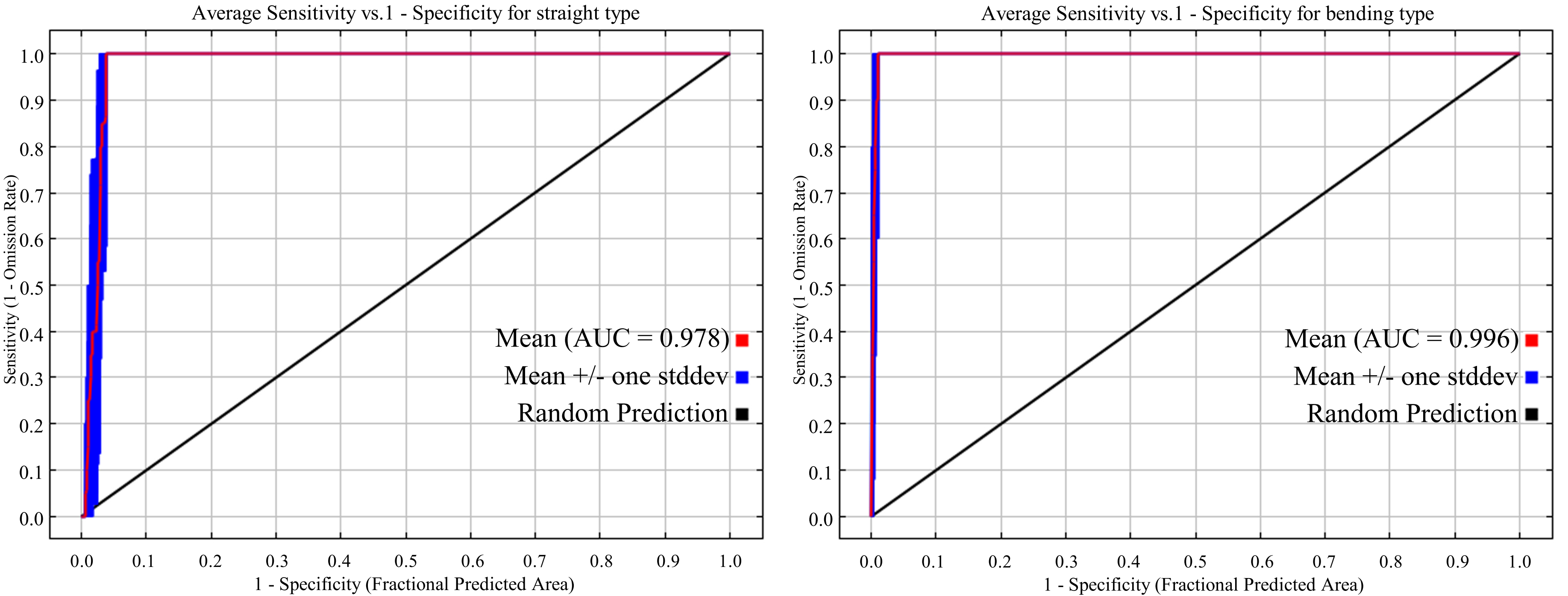

Average Sensitivity vs.1 - Specificity for straight type
Sensitivity (1 - Omission Rate)
1 - Specificity (Fractional Predicted Area)
0.0
0.1
0.2
0.3
0.4
0.5
0.6
0.7
0.8
0.9
1.0
Mean (AUC = 0.978)
Mean +/- one stddev
Random Prediction
Average Sensitivity vs.1 - Specificity for bending type
Sensitivity (1 - Omission Rate)
1 - Specificity (Fractional Predicted Area)
Mean (AUC = 0.996)
Mean +/- one stddev
Random Prediction
