# Supplementary figures and images for: Modeling the potential distribution of different types of Dendrocalamus sinicus, the strongest woody bamboo in the world, with MaxEnt model
Source: PeerJ. 2022 Aug 2;10:e13847. doi: 10.7717/peerj.13847 (PMC9354798; doi:10.7717/peerj.13847)

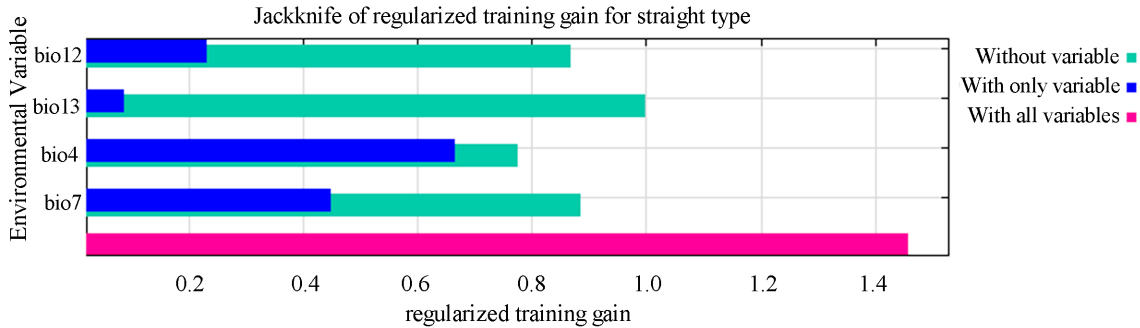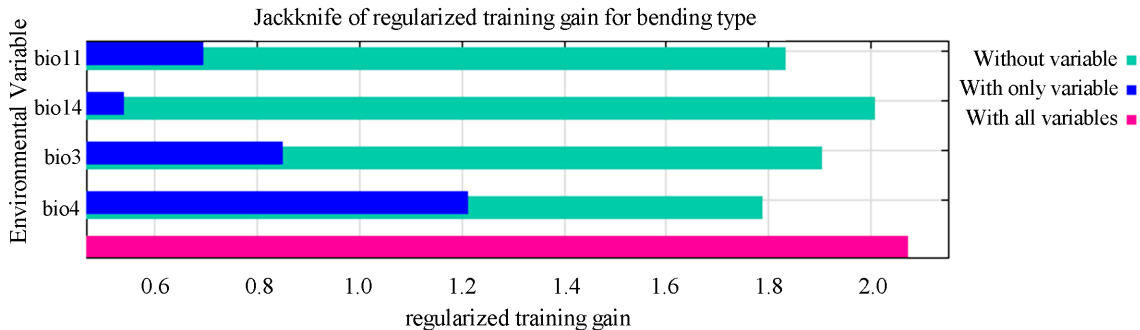

Supplement: Supplemental Information 2 [file peerj-10-13847-s002.pdf]

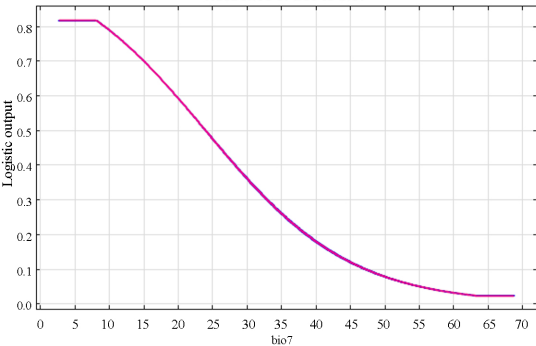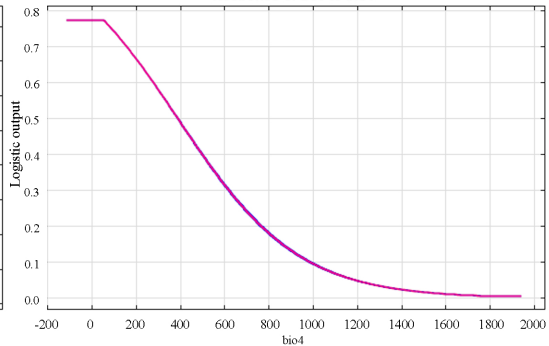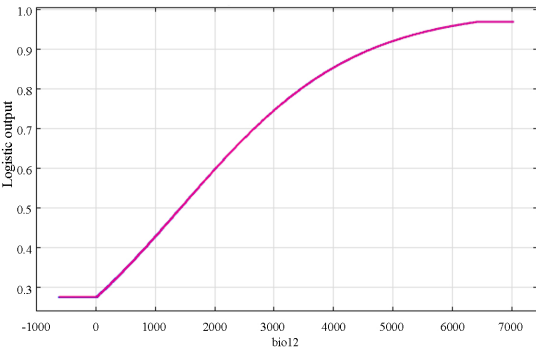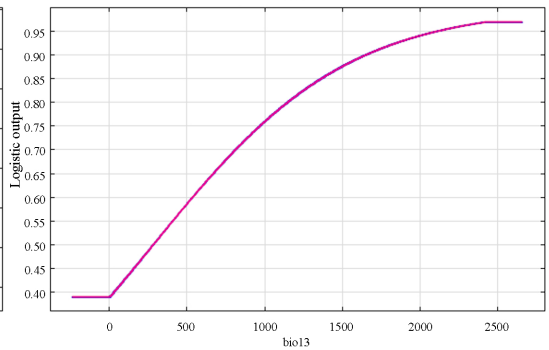

Supplement: Supplemental Information 3 [file peerj-10-13847-s003.pdf]

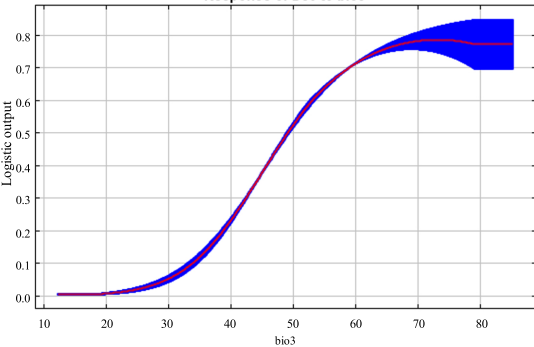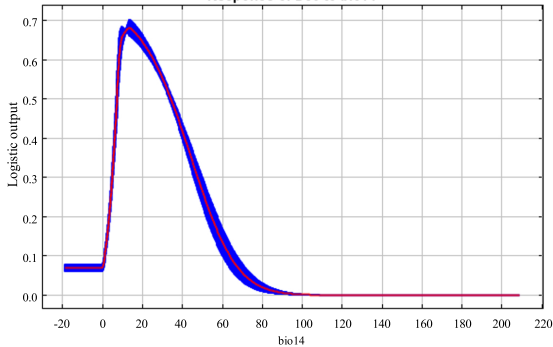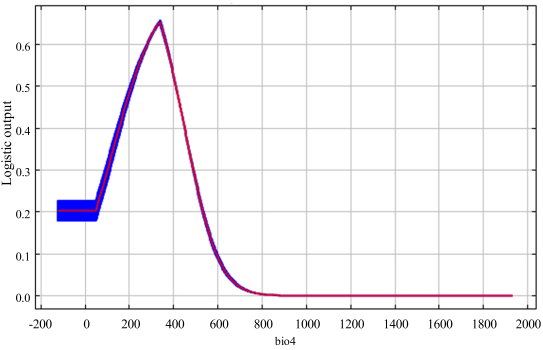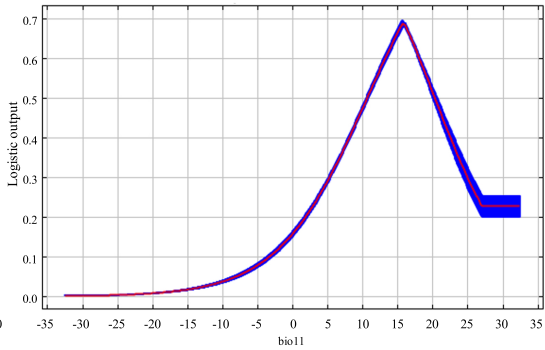

Supplement: Supplemental Information 4 [file peerj-10-13847-s004.pdf]
