# Supplementary material for: Modeling the potential distribution of different types of Dendrocalamus sinicus, the strongest woody bamboo in the world, with MaxEnt model
Source: PeerJ. 2022 Aug 2;10:e13847. doi: 10.7717/peerj.13847 (PMC9354798; doi:10.7717/peerj.13847)
Supplement: Supplemental Information 5 [file peerj-10-13847-s005.docx]

Table 1.19 bioclimatic variables

| Variable | Abbreviatn | Unit |
| --- | --- | --- |
| Annual Mean Temperature | bio1 | ℃ |
| Mean Diurnal Range (Mean of monthly (max temp - min temp)) | bio2 | ℃ |
| Isothermality (Bio2/Bio7) (×100) | bio3 | - |
| Temperature Seasonality (standard deviation *100) | bio4 | C of V |
| Max Temperature of Warmest Month | bio5 | ℃ |
| Min Temperature of Coldest Month | bio6 | ℃ |
| Temperature Annual Range (BIO5-BIO6) | bio7 | ℃ |
| Mean Temperature of Wettest Quarter | bio8 | ℃ |
| Mean Temperature of Driest Quarter | bio9 | ℃ |
| Mean Temperature of Warmest Quarter | bio10 | ℃ |
| Mean Temperature of Coldest Quarter | bio11 | ℃ |
| Annual Precipitation | bio12 | mm |
| Precipitation of Wettest Month | bio13 | mm |
| Precipitation of Driest Month | bio14 | mm |
| Precipitation Seasonality (Coefficient of Variation) | bio15 | C of V |
| Precipitation of Wettest Quarter | bio16 | mm |
| Precipitation of Driest Quarter | bio17 | mm |
| Precipitation of Warmest Quarter | bio18 | mm |
| Precipitation of Coldest Quarter | bio19 | mm |
