# Supplementary material for: Modeling the potential distribution of different types of Dendrocalamus sinicus, the strongest woody bamboo in the world, with MaxEnt model
Source: PeerJ. 2022 Aug 2;10:e13847. doi: 10.7717/peerj.13847 (PMC9354798; doi:10.7717/peerj.13847)
Supplement: Supplemental Information 8 [file peerj-10-13847-s008.docx]

| Distribution point | longitude | latitude |
| --- | --- | --- |
| 1 | 99.60861 | 22.44333 |
| 2 | 99.53861 | 22.31639 |
| 3 | 99.37944 | 22.22917 |
| 4 | 99.60139 | 22.16111 |
| 5 | 101.2517 | 21.93306 |
| 6 | 100.3444 | 21.85778 |
| 7 | 100.377 | 21.94987 |
| 8 | 101.5849 | 21.66172 |
| 9 | 101.6667 | 21.26667 |
| 10 | 100.2 | 21.75 |
| 11 | 100.05 | 21.7 |
| 12 | 100.3833 | 21.83333 |
