# Supplementary material for: Modeling the potential distribution of different types of Dendrocalamus sinicus, the strongest woody bamboo in the world, with MaxEnt model
Source: PeerJ. 2022 Aug 2;10:e13847. doi: 10.7717/peerj.13847 (PMC9354798; doi:10.7717/peerj.13847)
Supplement: Supplemental Information 9 [file peerj-10-13847-s009.docx]

| Distribution point | longitude | latitude |
| --- | --- | --- |
| 1 | 99.02056 | 23.50694 |
| 2 | 98.93806 | 23.44833 |
| 3 | 98.97528 | 23.37417 |
| 4 | 99.07778 | 23.32 |
| 5 | 98.98111 | 23.30889 |
| 6 | 99.09944 | 23.295 |
| 7 | 98.94722 | 23.25111 |
| 8 | 98.92972 | 23.22139 |
| 9 | 99.54139 | 22.72806 |
| 10 | 99.62222 | 22.63278 |
| 11 | 99.4675 | 22.51417 |
| 12 | 99.60861 | 22.44333 |
| 13 | 99.53861 | 22.31639 |
| 14 | 99.37944 | 22.22917 |
| 15 | 101.2517 | 21.93306 |
| 16 | 99.08333 | 23.56667 |
| 17 | 99.48333 | 22.75 |
